# Cdc42 subcellular relocation in response to VEGF/NRP1 engagement is associated with the poor prognosis of colorectal cancer

**DOI:** 10.1038/s41419-020-2370-y

**Published:** 2020-03-05

**Authors:** Li-li Ma, Li-li Guo, Yang Luo, Guang-long Liu, Yan Lei, Fang-yan Jing, Yun-li Zhang, Gui-hui Tong, Zhi-Liang Jing, Lan Shen, Min-shan Tang, Yan-qing Ding, Yong-jian Deng

**Affiliations:** 1 Department of Pathology, Nanfang Hospital and School of Basic Medical Sciences, Southern Medical University, 510515 Guangzhou, China; 2 Guangdong Provincial Key Laboratory of Molecular Tumor Pathology, 510515 Guangzhou, China; 3 Department of Pathology, Guang dong Provincial People’s Hospital, Guangdong Academy of Medical Sciences, 510515 Guangzhou, China; 4 Department of Pathology, First Affiliated Hospital, College of Clinical Medicine, Henan University of Science and Technology, 471000 Luoyang, Henan Province China; 5 Department of Urinary Surgery, the Fifth Affiliated Hospital of Southern Medical University, 510900 Guangzhou, China; 6 Department of Anorectal Surgery, Nanfang Hospital, Southern Medical University, 510515 Guangzhou, China; 7 Department of Oncology, Inner Mongolia Medical University, Hohhot, 010110 China; 8 Department of Pathology, General Hospital of Southern military Command, 510010 Guangzhou, China

**Keywords:** Cancer microenvironment, Targeted therapies

## Abstract

Microscopic indications of malignancy and hallmark molecules of cancer are pivotal to determining cancer patient prognosis and subsequent medical intervention. Here, we found that compared to apical expression of Cdc42, which indicated that basal expression of Cdc42 occurred at the migrating cell front, glandular basal expression of Cdc42 (cell division cycle 42) in tissues indicated poorer prognoses for colorectal cancer (CRC) patients. The current study shows that activated Cdc42 was rapidly recruited to the migrating CRC cell front after VEGF stimulation through engagement of membrane-anchored neuropilin-1 (NRP1). When VEGF signalling was blocked with NRP1 knockdown or ATWLPPR (A7R, antagonist of VEGF/NRP1 interaction), Cdc42 activation and relocation to the cell front was attenuated, and filopodia and invadopodia formation was inhibited. The VEGF/NRP1 axis regulates directional migration, invasion, and metastasis through Cdc42 activation and relocation resulting from actin filament polymerisation of the extensions of membrane protrusions. Collectively, the immuno-micromorphological pattern of subcellular Cdc42 at the cell front indicated aggressive behaviours and predicted poor prognosis in CRC patients. Disruption of the intra- and extracellular interactions of the VEGF/NRP1 axis or Cdc42 relocation could be performed in clinical practice because it might inhibit cancer cell motility and metastasis.

## Introduction

The search for morphological patterns or subcellular hallmarks to delineate the aggressive behaviours of cancer cells is still ongoing. For instance, a microscopic examination of filopodia and invadopodia can reveal the capacity for the high motility and invasiveness of cancer cells, but this procedure is inconvenient for conventional medical laboratories to perform on a regular basis. Light microscope imaging combined with determination of characteristic molecules related to malignant behaviour, i.e., immunostaining with specific antibodies, is generally accepted in current medical practice. Therefore, deeply deciphering the immuno-micromorphological pattern associated with migration, invasiveness, and metastasis is a requisite for the prognostic prediction of cancer.

Chemotaxis is the movement of cells towards specific sites in response to chemicals, and it is a central function of many cell types and plays a key role in cancer metastasis^1^. The migration directionality can be controlled through interactions between a tumour cell and its microenvironmental factors. Vascular endothelial growth factor (VEGF), including its variant VEGF165, in the microenvironment acts through an isoform-specific receptor, neuropilin-1 (NRP1), to attract the movement of cancer cells and is similar to neutrophil chemoattractant movements^2–5^. Our previous study showed that extracellular VEGF, not the intracellular VEGF of cancer cells, promoted the directional migration of colorectal cancer (CRC) cells, which improved the effectiveness of cell motility^3^. However, little is known regarding the subcellular transformation of VEGF/NRP1 engagement and its regulation of migrating cancer cells.

In filopodia, actin polymerisation pushes the plasma membrane forward^6^, whereas in invadopodia, actin polymerisation couples with degrading extracellular matrix (ECM) metalloproteases to clear a path for cell motility^7^, which guides the direction of migration^8^. Cell division cycle 42 (Cdc42) is known to regulate filopodia and invadopodia formation, cell polarity, invasion, directional migration, and secretion^9–13^. As a Rho GTPase family member, Cdc42 is active when GTP is bound and inactive when GDP is bound. Activated Cdc42 was shown to be important for persistent, unidirectional movement in EGF-mediated fibroblast motility^14^. Cdc42 overexpression is positively correlated with the histopathologic grade of CRC^15^, but the role of localised Cdc42 activation is poorly understood.

Many different molecules and signalling pathways coordinate the directional migration of cells, but the actin cytoskeleton and regulators of actin dynamics are involved in the formation of filopodia and invadopodia. Both VEGF and activated Cdc42 stimulate LIM domain kinase 2 (LIMK2) to phosphorylate and activate cofilin, which, in turn, regulates actin dynamics^16,17^. Because it has been demonstrated that VEGF controls the directionality of migrating CRC cells^3^, we propose that VEGF/NRP1 participates in the assembly of the cell front, such as the formation of filopodia and invadopodia and that Cdc42 might be involved in migration and invasion in response to VEGF stimulation, possibly uncovering a unique portrait of the molecular-subcellular frame.

The present study first revealed that the characteristic expression of Cdc42 in the CRC cell front was associated with poor prognosis in CRC patients. In vitro and in vivo models showed that Cdc42 activation and relocation resulted from VEGF/NRP1 engagement, which was related to the metastasis of CRC cells.

## Results

### The basal expression of Cdc42 is associated with poor prognosis of CRC

**Fig. 1:**
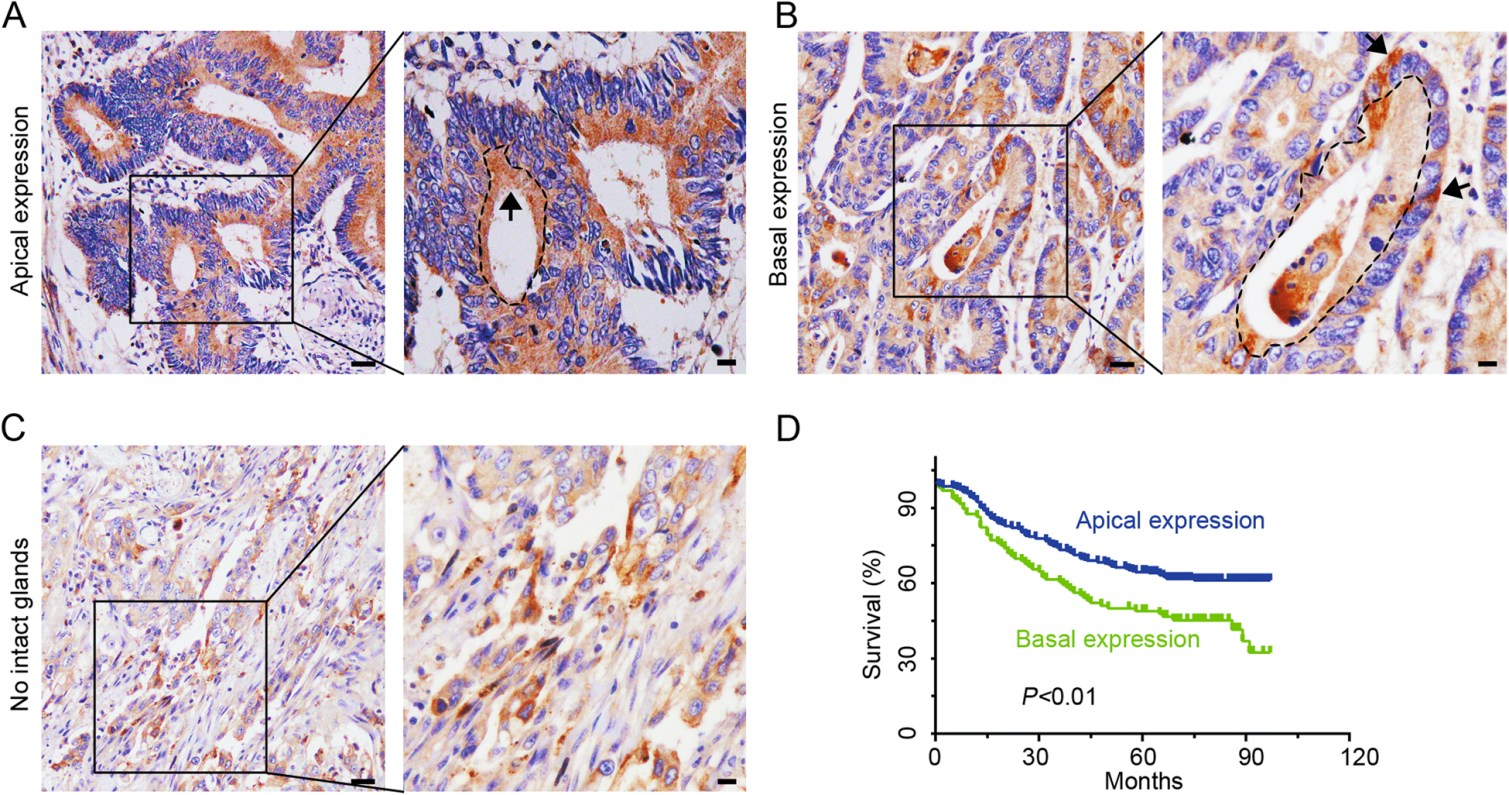
Cdc42 expression patterns as determined by immunohistochemical staining of CRC tissue. **a**, **b** CRC tissue with apical or basal expression. Arrows indicate the Cdc42 distribution, and the dotted curve represents the glandular cavity. Scale bars represent 50 or 10 μm. **c** Samples lacked intact glands in poorly differentiated adenocarcinoma tissue sections (scale bars: 50 μm, bars in enlarged images: 10 μm). **d** A Kaplan–Meier survival curve for CRC patients with Cdc42 expression in their tumour cells.

Aberrant Cdc42 expression or activation has been reported in CRC^15,18^. In the current study, IHC was used to examine Cdc42 distribution in 359 cases of well and moderately differentiated adenocarcinoma. The CRC tissue sections showed glandular apical or basal Cdc42 expression, which is referred to as “the glandular profile” (Fig. 1a, b). Although many cells in the poorly differentiated adenocarcinoma tissue sections showed polarised Cdc42 expression, no intact glands were available for referencing directionality (Fig. 1c), we excluded these cases for further analyses. Differences in apical and basal Cdc42 expression were found according to follow-up status (*P* < 0.01) and the presence of distant metastases (*P* < 0.001); however, no significant differences were detected for age, sex, differentiation, AJCC cancer stage, lymph node involvement, or tumour site (Table 1). The basal Cdc42 expression in the CRC cells implied that the cell front was infiltrating the interstitial tissues.

**Table 1:** Cdc42 with apical and basal distributions of CRC patients in association with clinicopathologic charcteristics (*n* = 359).

Characteristic	No. patients, %	Distribution of Cdc42		*P* Value
		Apical, *n* = 263 (%)	Basal, *n* = 96 (%)	
Gender				0.181
Male	204 (56.8)	155 (58.9)	49 (51.0)	
Female	155 (43.2)	108 (41.1)	47 (49.0)	
Age(years)				0.409
≤67	189 (52.6)	135 (51.3)	54 (56.3)	
>67	170 (47.4)	128 (48.7)	42 (43.7)	
Location				0.055
Colon	312 (86.9)	234 (89.0)	78 (81.3)	
Rectum	47 (13.1)	29 (11.0)	18 (18.7)	
Differentiation				0.325
Well	92 (25.6)	71 (27.0)	21 (21.9)	
Moderate	267 (74.4)	192 (73.0)	75 (78.1)	
AJCC cancer stage				0.226
I	39 (10.9)	33 (12.6)	6 (6.3)	
II	195 (54.3)	138 (52.5)	57 (59.4)	
III	116 (32.3)	84 (31.9)	32 (33.3)	
IV	9 (2.5)	8 (3.0)	1 (1.0)	
LN metastasis				0.787
Negative	247 (68.8)	182 (69.2)	65 (67.7)	
Positive	112 (31.2)	81 (30.8)	31 (32.3)	
Dis. metastasis				0.000
Negative	329 (91.6)	250 (95.1)	79 (82.3)	
Positive	30 (8.4)	13 (4.9)	17 (17.7)	
Status				0.001
Censored	204 (56.8)	163 (62.0)	41 (42.7)	
Death	155 (43.2)	100 (38.0)	55 (57.3)	

*LN* lymph node, *Dis. metastasis* distant metastasis.

Additionally, patients with basal Cdc42 expression had poorer prognoses than those who exhibited apical Cdc42 expression, as determined using Kaplan–Meier analysis based on a median follow-up of 73 months after radical surgeries (*P* < 0.01, Fig. 1d). Taken together, these results indicate that basal Cdc42 expression promotes progression in CRC patients.

### Extracellular VEGF stimulates intracellular Cdc42 activation and relocation

**Fig. 2:**
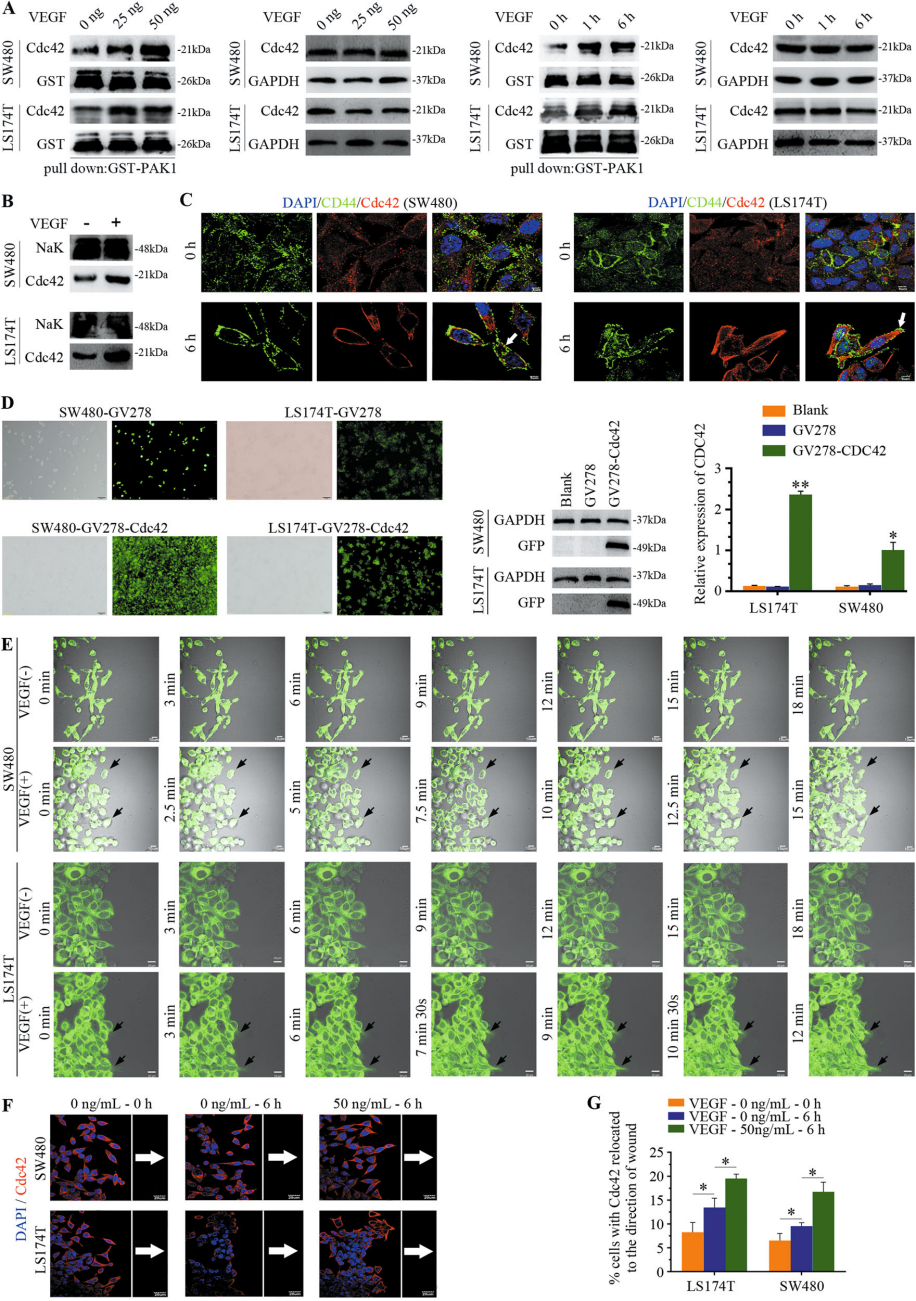
VEGF plays a critical role in the activation and localisation of Cdc42. **a** CRC cells were starved overnight in 1% FBS and then treated with either VEGF or not. Lysates were obtained, and Cdc42-GTP levels were assessed using the PAK1 pull-down assay. **b** Cell membrane fractions were isolated and probed for Cdc42. The NaK probe is a reference for membrane-bound proteins. **c** Cdc42/CD44 staining of SW480/LS174T cells. SW480/LS174T cells were treated as follows: SW480/LS174T cells were starved overnight in 1% FBS (VEGF-0 h); SW480/LS174T cells were starved overnight in 1% FBS and treated with 50 ng/mL VEGF for 6 h (VEGF-6 h). CD44 is a reference protein for the cell membrane. Scale bar, 5 μm. **d** Representative confocal image of CRC cells showing Cdc42 imaged with EGFP. Cdc42 protein expression was measured using western blotting, and Cdc42 was amplified using real-time quantitative polymerase chain reaction. Error bars represent the mean ± SD of triplicate experiments; **P < 0.01, *P < 0.05. Scale bar, 100 μm. **e**, **f** CRC cells were cultured to confluency on glass coverslips and then starved, scratched, and treated with VEGF. As shown in **e**, Cdc42 is located at the front of live CRC cells. Scale bar, 10 μm. Frames from typical time-lapse sequences are presented. Arrowheads denote the recognisable front of the cells at 15 min in SW480 cells and at 9 min in LS174T cells. Fixed Cdc42 cells were subjected to fluorescent immunostaining as shown in **f** (in red), and the nuclei were stained blue with [4′,6-diamidino-2′-phenylindole (DAPI). White arrows point to Cdc42 expression in cells oriented towards the scratched edge. The white reference line is parallel to the scratched edge and determines individual cell polarisation vs. the wound. Scale bar, 20 μm. **g** Quantitative analysis showed the cell percentages with Cdc42 expression oriented in the front of the cells; error bars represent the mean ± SD of triplicate experiments, *P < 0.001.

Based on the finding that VEGF can activate Cdc42 in endothelial cells^19^, we sought to determine the effect of VEGF on Cdc42 activation in CRC cells. Cdc42 activation was assessed by measuring Cdc42 pulldown levels using the p21-activated protein kinase PAK1, and this experiment revealed increased Cdc42 levels in CRC cells after extracellular VEGF stimulation compared with those in control cells (Fig. 2a). Additionally, membrane/cytosol fractionation and immunofluorescence staining indicated that intracellular Cdc42 translocated to CRC cell membranes following VEGF stimulation (Fig. 2b, c).

To investigate its intracellular localisation, Cdc42 was cloned into the GV278 vector using enhanced green fluorescent protein (EGFP) as a marker, and the corresponding GV278 vector without Cdc42 was used as the control. Cdc42 expression was detected using confocal microscopy, western blotting, and RT-PCR analyses (Fig. 2d). Wound healing assays showed that a few starved cells in the monolayers progressively extended pseudopodia with Cdc42-EGFP expression in the CRC cell front after VEGF stimulation (Fig. 2e). Importantly, Cdc42-EGFP expression at the cell front was enriched earlier in CRC cells with VEGF stimulation than in control cells, consistent with studies showing that activated Cdc42 promotes protrusion extensions^20^. We observed that the percentage of migrating CRC cells with Cdc42 enrichment at the cell front was significantly increased after VEGF stimulation (Fig. 2f, g). Overall, these results indicate that VEGF promotes the activation and relocation of Cdc42 in CRC cells.

### NRP1 engagement with VEGF is sufficient to activate and relocate Cdc42 to the CRC cell front

**Fig. 3:**
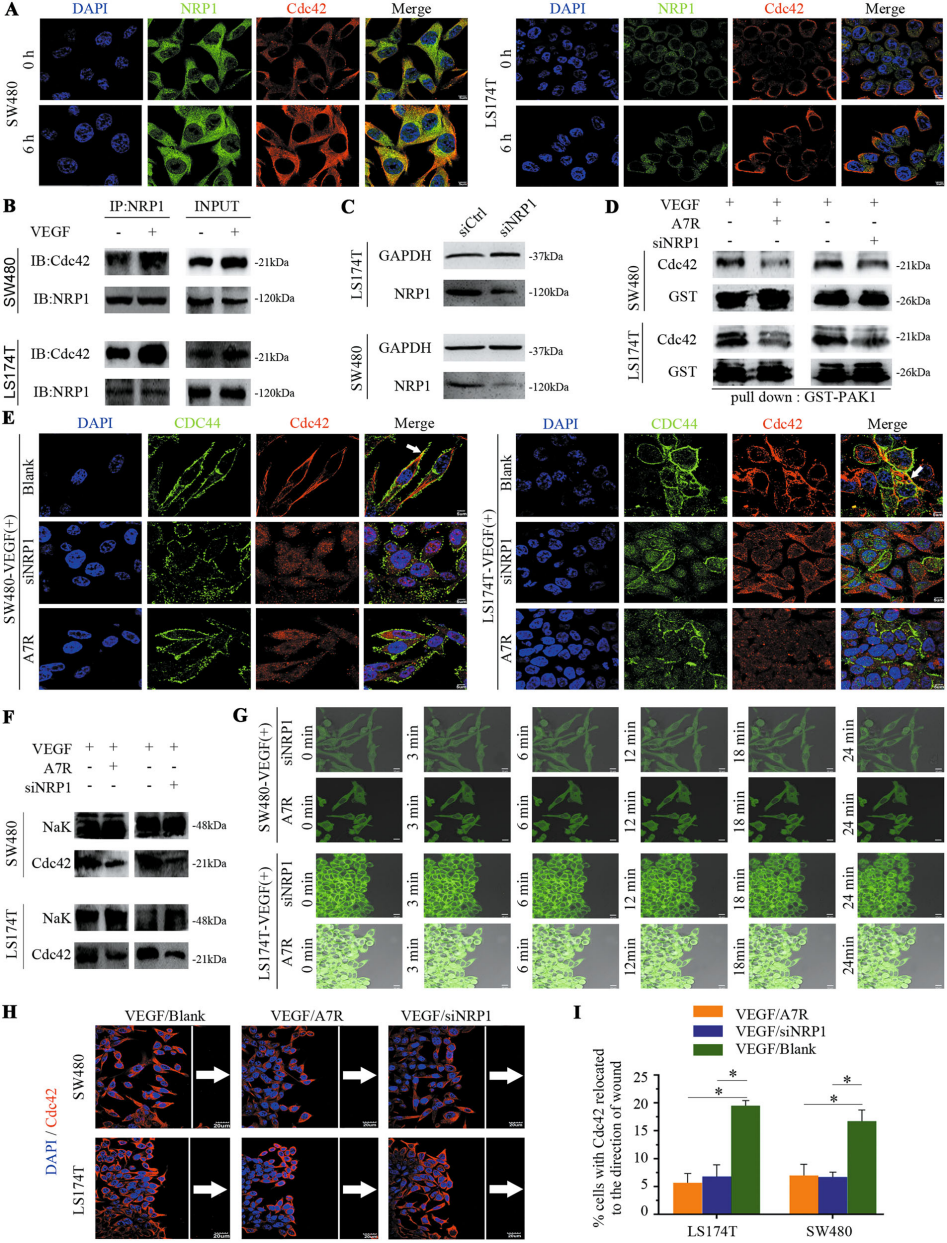
NRP1 is required for Cdc42 activation, and Cdc42 knockdown or A7R blockade impairs Cdc42 activation and relocation in CRC cells. **a** Cdc42 and NRP1 have dual staining in CRC cells. Scale bar: 5 μm. **b** NRP1 and Cdc42 are expressed in CRC cells with (+) or without (−) VEGF (50 ng/mL) treatment in the coimmunoprecipitation assay. **c** Western blot analysis of NRP1 in control (si-Ctrl) and NRP1 knockdown (si-NRP1) cells. **d** SW480/LS174T cells were transfected with control (−) or NRP1 siRNA (+) and then cultured with 50 ng/mL VEGF. SW480/LS174T cells were cultured with 50 ng/mL VEGF and either treated (+) or not (−) with A7R for 24 h. The lysates were obtained, and Cdc42-GTP levels were assessed using the PAK1 pull-down assay. **e** Cdc42/CD44-stained SW480/LS174T cells. SW480/LS174T cells were cultured with 50 ng/mL VEGF (VEGF/Blank); SW480/LS174T cells were cultured with 50 ng/mL VEGF mixed with A7R (VEGF/A7R or A7R); or SW480/LS174T cells were transfected with NRP1 siRNA and then cultured with 50 ng/mL VEGF (VEGF/siNRP1 or siNRP1). Scale bar: 5 μm. **f** Cell membrane fractions were isolated and probed for Cdc42. **g**, **h** SW480/LS174T cells were treated as follows: SW480/LS174T cells were cultured to confluency on glass coverslips, starved, scratched, and cultured with 50 ng/mL VEGF (VEGF/Blank) or with 50 ng/mL VEGF mixed with A7R (VEGF/A7R or A7R). Alternatively, SW480/LS174T cells were transfected with NRP1 siRNA and were cultured to confluency on glass coverslips, starved, scratched, and then cultured with 50 ng/mL VEGF (VEGF/siNRP1 or siNRP1). **g** shows Cdc42 localisation of the front in live CRC cells, scale bar, 10 μm. Frames from typical time-lapse sequences show SW480 and LS174T cells with unrecognisable protrusions even at 24 min. **h** shows fluorescent immunostaining of fixed Cdc42 (in red) and nuclei in blue (DAPI). White arrows point to Cdc42 in cells that are oriented towards the front. The white reference line is parallel to the scratched edge to determine individual cell polarisation vs. cells polarised towards the scratched edge. Scale bar, 20 μm. **i** Quantitative analysis shows the percentage of cells with Cdc42 oriented towards the front. Error bars represent the mean ± SD of triplicate experiments, *P < 0.001.

The VEGF-A isoform, VEGF165, acts through the NRP1 receptor in some tumour cells^21^. This receptor is upregulated in multiple tumour types^22^, supporting a role for NRP1 in tumour progression. Notably, Cdc42 is known to be activated by VEGF-A signalling in cultured endothelial cells and plays a critical role in actin remodelling in a variety of cell types, including cancer cells^23^. In the current study, immunofluorescence staining of CRC cells revealed that Cdc42 and NRP1 were colocalized in the cytoplasmic membrane. Moreover, immunofluorescence staining and coimmunoprecipitation analyses showed that NRP1/Cdc42 complexes were more prominent in the presence of VEGF than in its absence (Fig. 3a, b).

Cdc42 location and activation in the CRC cell front after VEGF stimulation raised the possibility that VEGF promotes Cdc42 relocation or activation via NRP1. To investigate whether NRP1 is indeed responsible for VEGF-dependent Cdc42 activation and relocation, we knocked down NRP1 expression (Fig. 3c) and blocked VEGF/NRP1 interactions with the antagonist ATWLPPR (A7R)^24^. Cdc42 activation levels (Fig. 3d) and membrane accumulation were reduced (Fig. 3e, f) in NRP1-silenced and A7R-treated cells, even in the presence of VEGF stimulation. Scratch wound assays revealed that the ability of Cdc42 to translocate to the front was dramatically attenuated in NRP1-silenced cells and those supplemented with A7R, even in the presence of VEGF stimulation (Fig. 3g–i). Taken together, our data reveal that NRP1 plays a key role in activating and translocating Cdc42 to the cell membrane.

### VEGF/NRP1-activated Cdc42 induces the formation of filopodia in CRC cells

**Fig. 4:**
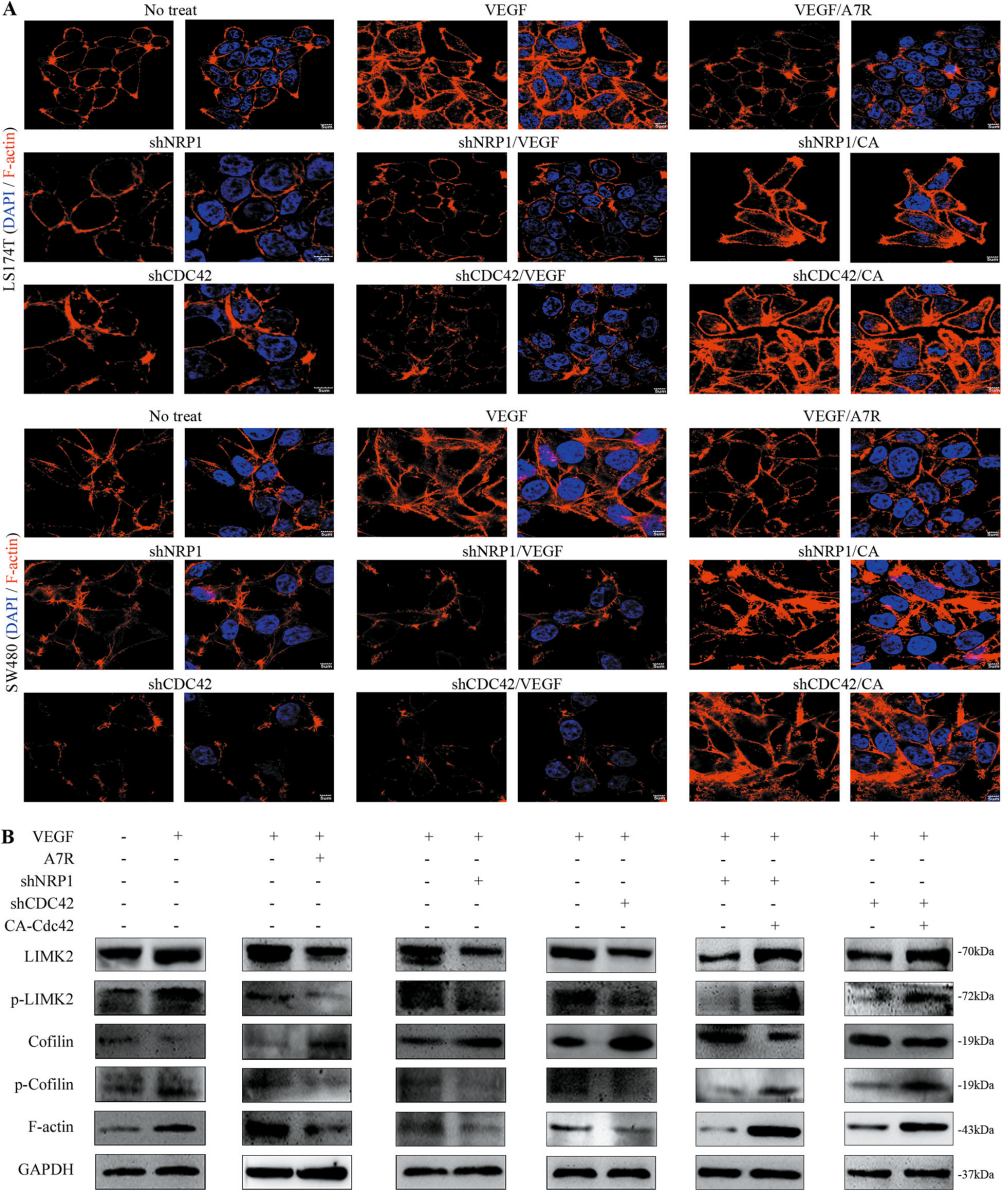
Cdc42 regulates F-actin aggregation in the cell front of CRC cells. **a** Immunofluorescence images of F-actin expression in CRC cells. Scale bar, 5 μm. CA = Cdc42V12, the constitutively active form of Cdc42. **b** Western blotting analysis shows LIMK2, p-LIMK2, cofilin, p-cofilin, and F-actin expression in CRC cells cultured in different conditions.

Cdc42 was found to activate LIMK2 and subsequently phosphorylate cofilin, which thereby promoted F-actin formation for initiation of filopodia formation^16^, a critical early step needed for guiding cell migration^25^. We found that in response to VEGF stimulation, intracellular F-actin expression increased in the CRC cell front and was attenuated after NRP1/Cdc42 knockdown or A7R treatment. This effect was completely reversed in cells that overexpressed Cdc42V12 (the constitutively active form of Cdc42, CA)^16^ (Fig. 4a, b).

We next observed that cofilin (p-cofilin) and LIM-kinase (p-LIMK) phosphorylation was increased in CRC cells stimulated with VEGF compared with the other groups (the NRP1/Cdc42 knockdown and A7R-conditioned media groups; Fig. 4b). Cdc42V12 returned p-cofilin expression to VEGF-stimulated levels (Fig. 4b). The effects of VEGF-stimulated Cdc42 activation on the LIMK2/cofilin/F-actin cytoskeleton suggest that Cdc42 is specifically involved in the cytoskeletal dynamics necessary for CRC cell directional migration.

### VEGF/NRP1-activated Cdc42 additionally promotes invadopodia formation by degrading gelatine

**Fig. 5:**
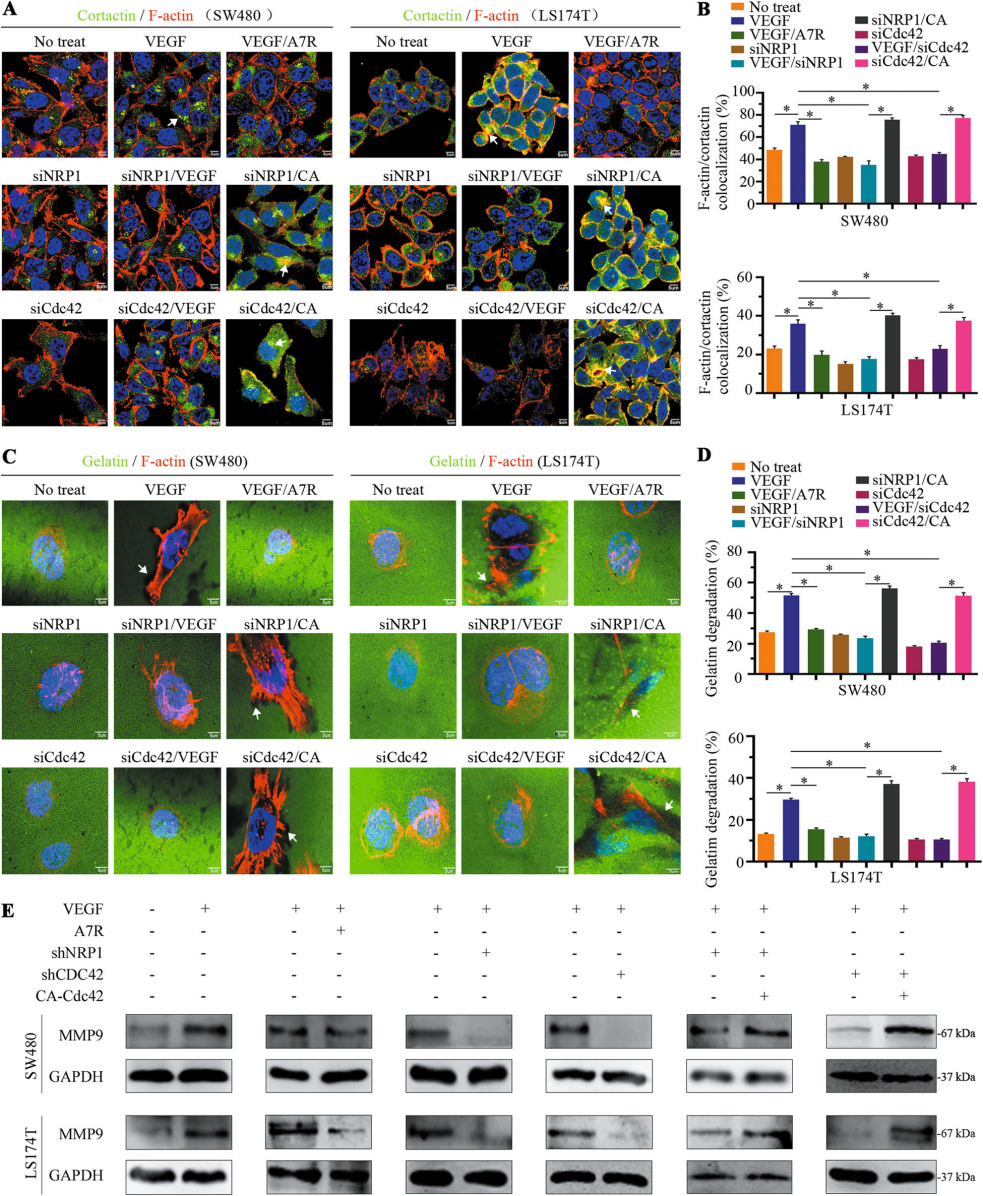
Cdc42 is activated by the VEGF/NRP1 axis, which is associated with invadopodia formation in the cell front. **a** Immunofluorescence analysis showing F-actin (red) and cortactin (green) colocalisation in SW480 and LS174T cells. Arrows indicate colocalization of F-actin and cortactin in the cytoplasm. Scale bar, 5 μm. **b** The percentage of SW480 and LS174T cells with colocalized F-actin/cortactin was quantified. Data were derived from eight random fields and are expressed as the mean ± SD, *P < 0.001. **c** Treated cells were seeded onto Oregon Green 488-conjugated gelatin-coated coverslips for 24 h and then stained with an F-actin antibody (red). Arrows indicate the black region of gelatine degradation. Scale bar, 5 μm. **d** The percentage of gelatine degradation was quantitatively analysed. Data were derived from six random fields and are expressed as the mean ± SD, *P < 0.001. **e** The level of MMP9 in CRC cells cultured in different conditions.

Invadopodia are actin-rich structures found in invasive cancer cells that are enzymatically active and degrade the surrounding ECM to facilitate invasion^26^. CRC cells were co-stained with F-actin and cortactin, which are often used as indicators of invadopodia precursors^8^. In VEGF-treated cells, we found that some colocalised F-actin/cortactin formed punctate-like structures in the cytoplasm, which implied the presence of an invadopodia precursor. However, in cultured cells with Cdc42/NRP1 knockdown or A7R-conditioned media, F-actin/cortactin colocalization in the cytoplasm was weak, even after VEGF treatments. The colocalization ability was restored when Cdc42V12 was overexpressed in these cultures (Fig. 5a, b).

Invadopodia are membrane protrusions with enzymes required for ECM degradation. To further verify that the VEGF/NRP1 axis influenced the function of invadopodia through Cdc42 activation, we used an Oregon Green 488-conjugated gelatine degradation assay to monitor invadopodia activity. As shown in Fig. 5c, the F-actin that accumulated in the cytoplasm confronted black regions from the degraded gelatine of the VEGF-treated cells. However, F-actin accumulation in CRC cells accompanied by gelatine degradation was blocked in cells with Cdc42/NRP1 knockdown or A7R-conditioned media, even in the presence of VEGF treatments. Conversely, the ability to degrade gelatine was regained in cells with Cdc42V12 overexpression (Fig. 5c, d), and these changes were a consequence of changed matrix metalloproteinase 9 (MMP9) expression and activity (Fig. 5e). These data indicate that the VEGF/NRP1 axis plays a crucial role in invadopodia formation and ECM degradation to assist in directional migration.

### VEGF/NRP1-mediated Cdc42 relocation promotes the migration, invasion, and metastasis of CRC cells

**Fig. 6:**
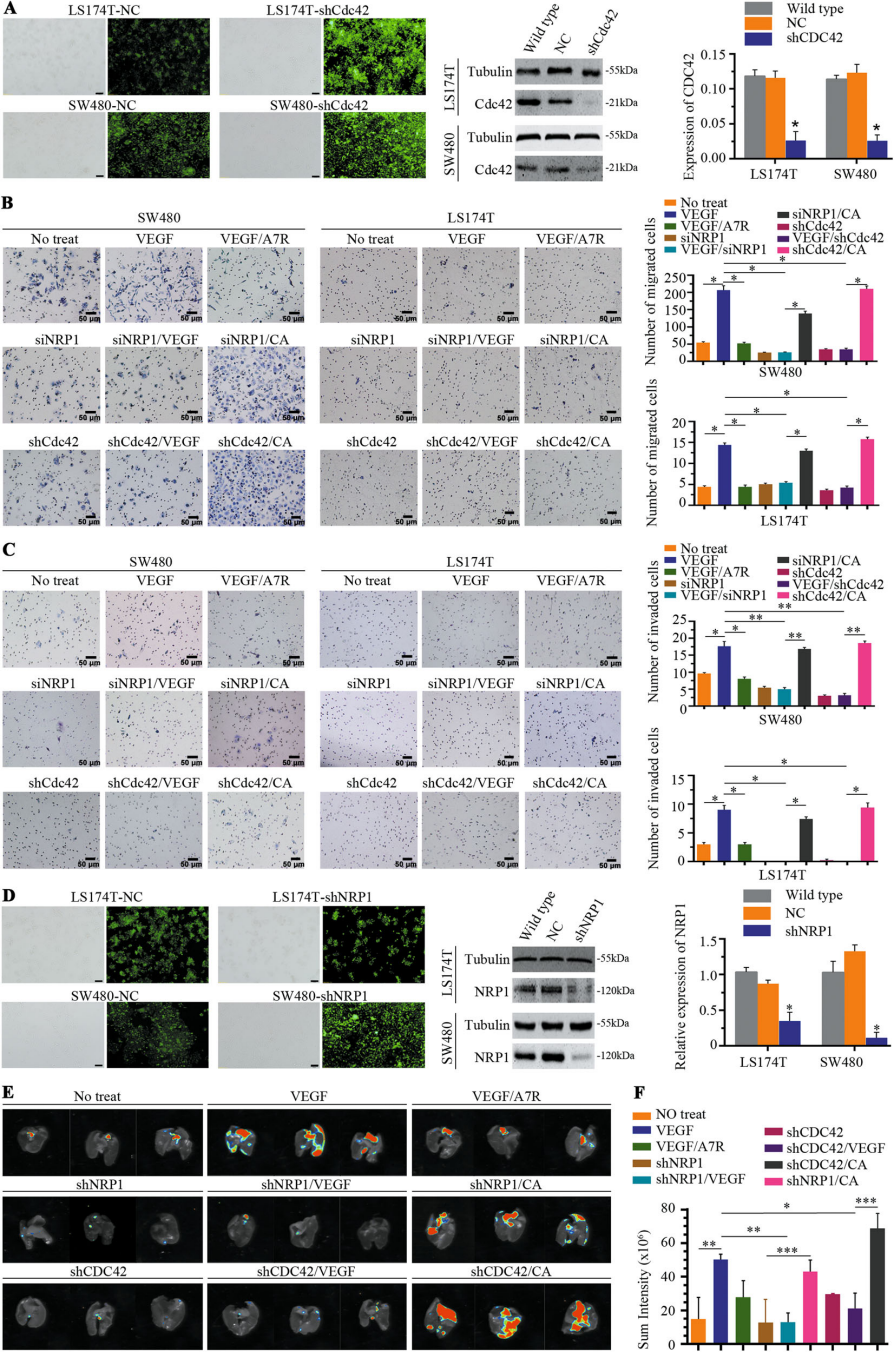
Activated Cdc42 promotes migration/invasion and lung metastases of CRC cells. **a** LS174T and SW480 cells were transfected with NC or shCdc42 in the first column, scale bar, 100 μm, and Cdc42 expression was detected using western blotting in the second column. Cdc42 was amplified using real-time quantitative polymerase chain reaction in the third column. NC represents cells infected with empty lentiviruses and was used as a negative control. Error bars represent the mean ± SD of triplicate experiments; *P < 0.001. **b** Transwell migration activity of SW480 and LS174T cells induced by different conditions and representative images. Data are the means ± SD from three independent experiments, *P < 0.001. Scale bar, 50 μm. **c** Representative images of the Transwell invasion assay showed that SW480 and LS174T cells were induced by different conditioned media. Data are presented as the means ± SD from three independent experiments, *P < 0.01, **P < 0.001. Scale bar, 50 μm. **d** LS174T and SW480 cells were transfected with NC or shNRP1 in the first column, scale bar, 100 μm, and NRP1 expression was detected using western blotting in the second column. NRP1 was amplified using real-time quantitative polymerase chain reaction in the third column. NC represents the cells infected with empty lentivirus and was used as a negative control. Error bars represent the mean ± SD of triplicate experiments; *P < 0.001. **e** Representative bioluminescent images of nude mouse lungs 30 days after intravenous injection. **f** Quantification analysis of fluorescence signals from captured bioluminescence images. *P < 0.05, **P < 0.01 and ***P < 0.001.

To test whether the VEGF/NRP1 axis promotes the migration and invasion of CRC cells through Cdc42 activation, we knocked down Cdc42 (Fig. 6a) and performed wound healing and Transwell assays following VEGF stimulation. The SW480 migration distance increased following treatment with VEGF for 48 h, as was seen in LS174T cells after incubation with VEGF for 96 h (Supplementary Fig. 1A, B). However, after knockdown of NRP1 (Fig. 3c) or Cdc42 (Fig. 6a) or supplementation with A7R-conditioned media, cell migration distances were significantly reduced (Supplementary Fig. 1A, B).

We next performed Transwell assays to verify the involvement of Cdc42 in VEGF-induced CRC cell migration and invasion in vitro. Compared to the control group (without VEGF stimulation of LS174T or SW480 cells, no treatment group), more SW480 and LS174T cells migrated/invaded to the lower chamber after VEGF stimulation, whereas cells with NRP1/Cdc42 knockdown or in A7R-conditioned media significantly inhibited the migration/invasion capacity (Fig. 6b, c) with VEGF treatment. The migration/invasion ability was restored when Cdc42V12 was overexpressed. These results confirm that VEGF-mediated Cdc42 activation plays an important role in tumour cell migration in response to VEGF stimulation.

We next sought to verify our in vitro findings regarding the effect of VEGF stimulation on CRC cell migration and invasion in vivo. LS174T and SW480 cells were infected with shNRP1 via lentiviral infection to establish stable NRP1 knockdown lines; negative control (NC) cell lines were transduced with empty lentiviral particles (Fig. 6d). We then confirmed the effectiveness of NRP1 knockdown in LS174T and SW480 cells (Fig. 6d). These cells were intravenously injected into the tail veins of immunocompromised mice, and bioluminescence imaging was used to track lung implantation. The group receiving VEGF-stimulated cells exhibited significantly stronger fluorescence signals than the control group, indicating that VEGF stimulation increased CRC lung metastasis. NRP1 or Cdc42 silencing attenuated VEGF-induced fluorescent signals. Moreover, Cdc42V12-GFP transfection dramatically rescued the ability of CRC cells to migrate in the context of wild-type NRP1 or Cdc42 silencing (Fig. 6e, f). Taken together, our data demonstrate that the VEGF/NRP1 axis promotes CRC cell metastasis by controlling downstream Cdc42 activation, both in vitro and in vivo.

## Discussion

Distant metastases predict poorer prognoses for cancer patients and are responsible for the majority of CRC-related deaths. Cancer cell migration is the first step of metastasis and is a directional rather than a random process^27^. Remodelling of agents and intracellular molecules in the microenvironment is required to efficiently guide directional migration. Our previous study revealed that VEGF was a key microenvironmental agent that controls the directionality of migrating CRC cells^3^; however, the molecules that are involved in the assembly and regulation of cancer cell directional migration, such as chemokines and agents guiding directionality, remain largely unknown^28^. The current study further showed that VEGF/NRP1 engagement mediates Cdc42 activation and relocation for efficient migration, invasiveness, and metastasis.

Directional migration requires sustained forward movement of the plasma membrane at the leading edge of migrating cells, where filopodia and invadopodia occur^29^. Filopodia command the direction of the migrating cells^6^ and invadopodia are ECM-degrading structures that arise on the ventral surface of cell membranes^7^. Activated Cdc42 is essential to initiate a variety of cellular responses, including cell invasion, migration, invadopodia and filopodia formation, and cell polarity^9,10^. Although a previous study reported that Cdc42 was activated after wound initiation and localised to the plasma membrane at the leading edge of migrating cells^30^, the agents involved in the relocation of Cdc42 and the control of secondary effects are poorly understood.

NRP1 was found to be involved in Cdc42 activation, in which primary human endothelial cell migration was inhibited by Cdc42 or NRP1 knockdown^31^. This study first found that Cdc42 was expressed in the basal locations of glandular CRC tissue sections, which facilitated the infiltration of CRC cells through interstitial tissues. Therefore, we focused on the potential role of Cdc42 activation in VEGF/NRP1-induced directional migration of CRC cells. The data demonstrated that NRP1 was necessary for Cdc42 activation and relocation using VEGF stimulation. Then, in subsequent experiments, activated Cdc42 altered not only LIMK2 and cofilin phosphorylation in CRC cells but also reduced filamentous actin (F-actin) depolymerisation. Additionally, filopodia and invadopodia formation at the CRC cell front were dependent on Cdc42 activation that resulted from VEGF/NRP1 signals.

Activated Cdc42 in the cell front is known to regulate cytoskeletal organisation and metalloproteinase expression and activation, which are important for directional migration and ECM degradation^16,30,32^. LIMK2 is one of the Cdc42 targets that is involved in this process and regulates cofilin-mediated actin depolymerisation^33^. Filopodia and invadopodia are F-actin-based membrane protrusions^34,35^. Invadopodia degrade the ECM and weaken the ability of cells to resist movement. Collectively, Cdc42 activation and relocation promote F-actin enrichment at the front and ECM degradation by invadopodia in migrating CRC cells, which further promotes directional migration. Therefore, VEGF/NRP1 engagement guides the directionality of CRC cell migration.

The nude mouse model further revealed that CRC pulmonary metastases were associated with VEGF stimulation, NRP1 transfer, and Cdc42 activation, which corroborated the findings of our previous study and indicated that the directionality of protrusion formation and the high VEGF levels in interstitial tissues were consistent with metastatic production^3^. Cdc42 activation and relocation are at the core of the cell front assembly, which involves filopodia and invadopodia. Basal Cdc42 expression in the CRC tissues was equal to the front, directing CRC cells to migrate into the interstitial tissues. Thus, it is likely that basal Cdc42 expression is related to aggressive CRC behaviour in patients with poor prognoses.

**Fig. 7:**
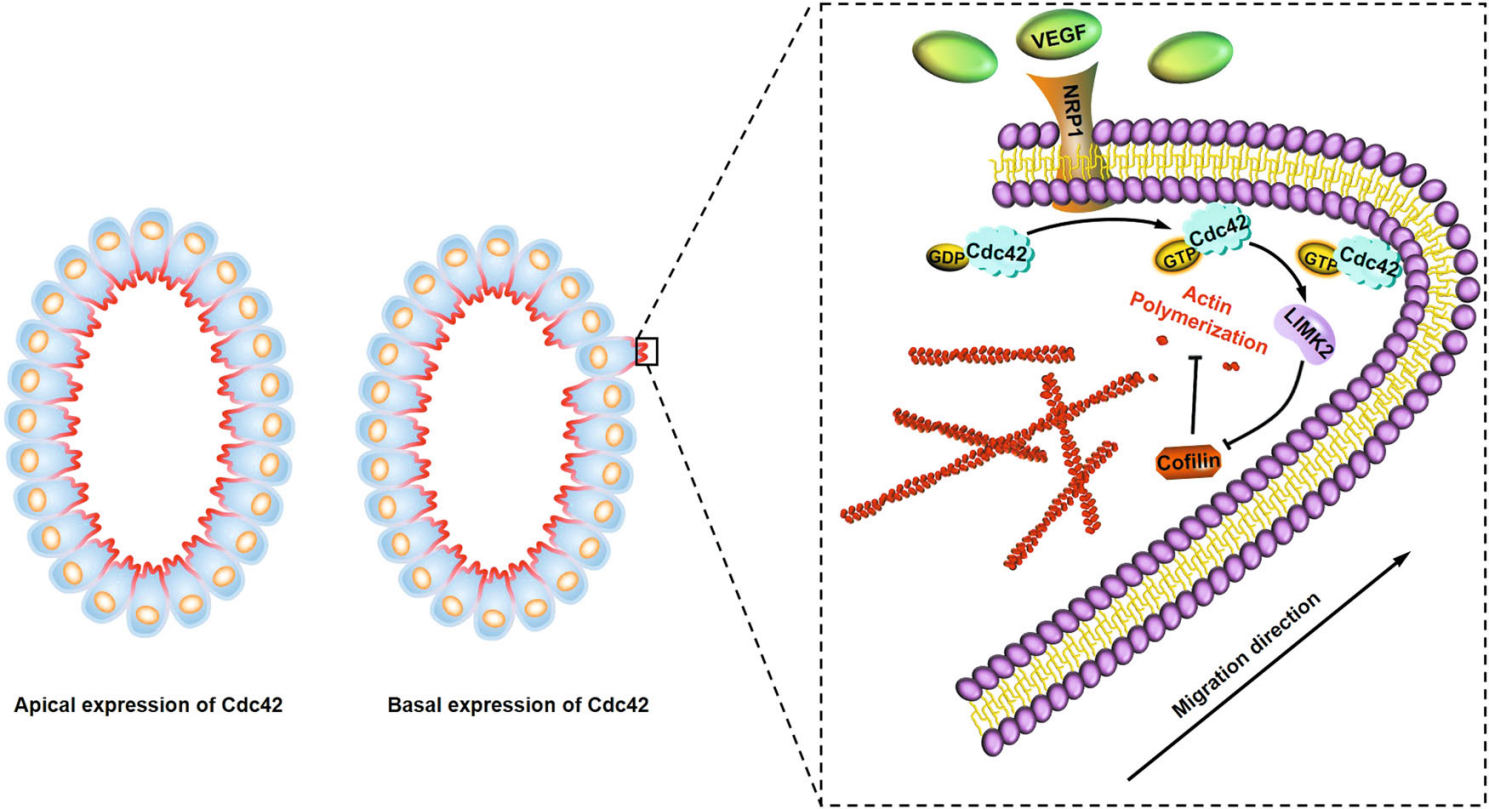
Schematic of Cdc42 expression predicting the prognosis of colorectal cancer. VEGF/NRP1 engagement activates and relocates Cdc42 to the migrating CRC cell front, which controls actin filament polymerisation of the extensions of membrane protrusion and the directionality of migration.

In conclusion, this study revealed that VEGF/NRP1 signalling activated and relocated Cdc42, which resulted in extension of the membrane protrusion and directional migration of CRC cells with critical enhancement of the metastatic potential. In other words, blocking the VEGF/NRP1 axis could be a therapeutic strategy used to prevent metastatic spread of CRC in clinical settings (Fig. 7).

## Materials and methods

### Patient tissues

Tumour tissues were obtained from 72 patients (Nanfang Hospital of Southern Medical University, Guangzhou, China) and 287 patients (Outdo Biotech, Shanghai, China). Patients or relatives approved the use of clinical materials for research purposes according to the Ethics Committee of Southern Medical University. Clinical samples were immunostained with a Cdc42 antibody (1:100; ab64533, Abcam). Immunohistochemistry (IHC) staining was performed with the EnVision Detection System (K500711, DAKO Corporation, Copenhagen, Denmark). The IHC results for Cdc42 were evaluated using this method: 10 high magnification fields were examined for each sample, and if basal expression or both basal and apical distributions were observed in any field of a given sample, we defined this case as basal distribution. If only apical distribution was observed in any field, then this case was defined as apical distribution.

### Cell culture

Colorectal cancer cell lines LS174T and SW480 were obtained from the American Type Culture Collection (ATCC, Manassas, VA, USA) and propagated using ATCC recommendations. All cell lines were verified by STR DNA profiling analysis, and monitoring for mycoplasma contamination was performed using a Mycoplasma Stain Assay Kit. The CRC cell lines were cultured in RPMI 1640 medium supplemented with 10% foetal bovine serum (FBS) (Gibco, Paisley, UK) at 37 °C in a humidified incubator at 5% CO_2_. VEGF was purchased from PeproTech (Cat#100-20, PeproTech, Rocky Hill, NJ, USA).

### Animal model assays

Animal experiments were reviewed and approved by the Institutional Animal Care and Use Committee at our university. Female nude mice, 4–6-weeks-old, were purchased from and maintained at the Central Laboratory of Animal Science at Southern Medical University. Three mice per group were randomly divided. Treated cells were suspended in phosphate-buffered saline (PBS) and injected intravenously via the tail vein. Every 3 days, VEGF was injected into mice from the VEGF stimulation group. Thirty days after the last injection, the incidence of metastases in mice was estimated using bioluminescence and a whole-body green fluorescent protein (GFP) imaging system (FX Pro, Bruker, Billerica, MA, USA). The signal level was used to assess the relative tumour burden in mouse lungs.

### Cdc42 pull-down, immunoprecipitation, and immunoblotting assays

To detect Cdc42 activation, the Cdc42 Activation Detection Kit (Cell Signalling Technology, Beverly, MA, USA) was used according to the manufacturer’s instructions. Briefly, GTP-bound Cdc42 was isolated with glutathione-agarose beads bound to the PAK1 p21-binding domain via a glutathione S-transferase (GST) tag and was then identified by immunoblotting of the eluted proteins with a Cdc42 antibody.

### Isolation of plasma membranes

Plasma membrane extracts were prepared using the Membrane and Cytosol Protein Extraction Kit (Beyotime, Haimen, China). Plasma membranes were extracted according to the manufacturer’s instructions, and the plasma membrane fractions were tested for the plasma membrane marker ATP1B1 (1:500, A5793, ABclonal).

### Immunofluorescence assay

CRC cells were fixed in 4% paraformaldehyde and incubated with Cdc42 (1:100; ab64533, Abcam)/NRP1 (1:100, 60067-1-Ig, ProteinTech) or F-actin (5 μg/mL, 4E3.adl, Abcam) antibodies overnight at 4 °C. The cells and sections were stained with secondary antibodies (1:100, Zhongshan Golden Bridge Biotechnology), the nuclei were detected with 4′,6-diamidino-2-phenylindole (DAPI, Sigma), and the cells were then counted and examined using confocal microscopy (FV1000, Olympus).

### Stable transfection and intracellular Cdc42 localisation

Lentivirus/GV278-CDC42 (Ubi-EGFP-MCS-IRES-puromycin) and the corresponding control lentivirus/GV278 vectors were obtained from GeneChem (Shanghai, China) and used for transfection according to the manufacturer’s protocol.

### Knockdown of Cdc42 and NRP1 by lentiviral vectors

To establish a stable knockdown of Cdc42 and NRP1, SW480 and LS174T cells were infected with lentiviruses expressing shRNA against Cdc42 and NRP1. The shRNA was designed and packaged by GeneChem (Shanghai, China). The sequences of the two cDNA fragments are as follows: NRP1,

5′-CAGATCACAGCTTCTTCCCAGTATA-3′; and CDC42, 5′-AAAGACTCCTTTCTTGCTTGT-3′. Cell transfections with the virus were performed according to the manufacturer’s instructions.

### Small interfering RNAs (siRNAs)

siRNAs against NRP1 (5′-GGACAGAGACTGCAAGTAT-3′) or CDC42 (5′-AAAGACTCCTTTCTTGCTTGT-3′) were purchased from Ribo-bio (Guangzhou, China) and were manipulated according to the manufacturer’s protocol. Cdc42 levels were detected using the protocol described in our previous study^3^.

### Immunofluorescence staining of injured cells

Treated CRC cells were seeded in each well of a six-well plate, allowed to reach confluency, and then starved overnight in 1% FBS. Treated cells were then trypsinised and counted. Approximately 5 × 10^5^ cells were reseeded in each well of a new six-well plate, and confluent cell monolayers were scratched with a 10-μL sterile pipette tip. Then, the non-adherent cells were washed away with sterilised PBS, and the media was replaced with either starvation media, RPMI 1640 with 1% FBS and containing VEGF, or RPMI 1640 with 1% FBS and containing VEGF/A7R. The cells were then allowed to migrate. Next, immunofluorescent staining was performed, and cells were counted and examined using confocal microscopy (FV1000, Olympus).

### Monitoring the repair of scratched cell monolayers

After the cells were scratched and washed with PBS, serum-free media was added to the wells, and the gap areas were monitored using an inverted microscope (Olympus, Japan). Three random non-overlapping areas in each well were captured at 0, 48 (SW480), and 96 h (LS174T) post-scratch. The scratch width between the two linear regions was quantitated by assessing the capacity of cells to migrate.

### Transwell migration and invasion assays

CRC cells (2 × 10^5^) were seeded in a Transwell insert (8-μm filters, Corning) coated with Matrigel (invasion assays, BD Biosciences, La Jolla, CA, USA) or uncoated (migration assays) for different periods of time. After incubation, cells were fixed with 4% paraformaldehyde for 10 min. The cells that did not migrate were removed with a cotton swab, stained with haematoxylin, and imaged using an inverted microscope. The data were derived from six random pictures.

### ECM degradation assays

Treated CRC cells were seeded on Oregon Green 488-conjugated gelatine (0.2 mg/ml, G13186, Invitrogen)-coated glass coverslips containing a thin layer of gelatin. After a 24 h incubation, cells were fixed, permeabilized, and stained with F-actin. Cells were counted and examined using confocal microscopy (FV1000, Olympus). At least 100 cells were quantified for each condition and cell line.

### Statistical analysis

Each assay was performed in triplicate and independently repeated at least three times. The results are expressed as the mean ± SD. The data were analysed by Student’s *t*-test or one-way analysis of variance (ANOVA) to determine statistical significance. Pearson’s *χ*^2^ test or Fisher’s exact test was used to analyse the relationship between Cdc42 expression and the clinicopathologic features. Survival curves were obtained using the Kaplan–Meier method. All analyses were two-sided and conducted using SPSS version 13.0 software for Windows; values of *P* < 0.05 were considered statistically significant.

## Supplementary information

Supplementary figure legends

Supplemental Fig. 1

Author Contribution Form
