# Supplementary material for: Cdc42 subcellular relocation in response to VEGF/NRP1 engagement is associated with the poor prognosis of colorectal cancer
Source: Cell Death Dis. 2020 Mar 5;11(3):171. doi: 10.1038/s41419-020-2370-y (PMC7058620; doi:10.1038/s41419-020-2370-y)
Supplement: Supplementary file 1 — Supplementary figure legends [file 41419_2020_2370_MOESM1_ESM.docx]

# Supplementary figure legends

**Supplementary Fig. 1. Cdc42 was activated by VEGF, promoting the migration of CRC cells**

**(A)** Representative wound healing images were obtained at different times. Scale bar, 100 μm.

**(B)** The relative distances between wound edges of CRC cells at different times. Error bars represent the mean ± SD of triplicate experiments; ***P*  < 0.001, **P*  < 0.01.
