# Supplementary figures and images for: Cdc42 subcellular relocation in response to VEGF/NRP1 engagement is associated with the poor prognosis of colorectal cancer
Source: Cell Death Dis. 2020 Mar 5;11(3):171. doi: 10.1038/s41419-020-2370-y (PMC7058620; doi:10.1038/s41419-020-2370-y)

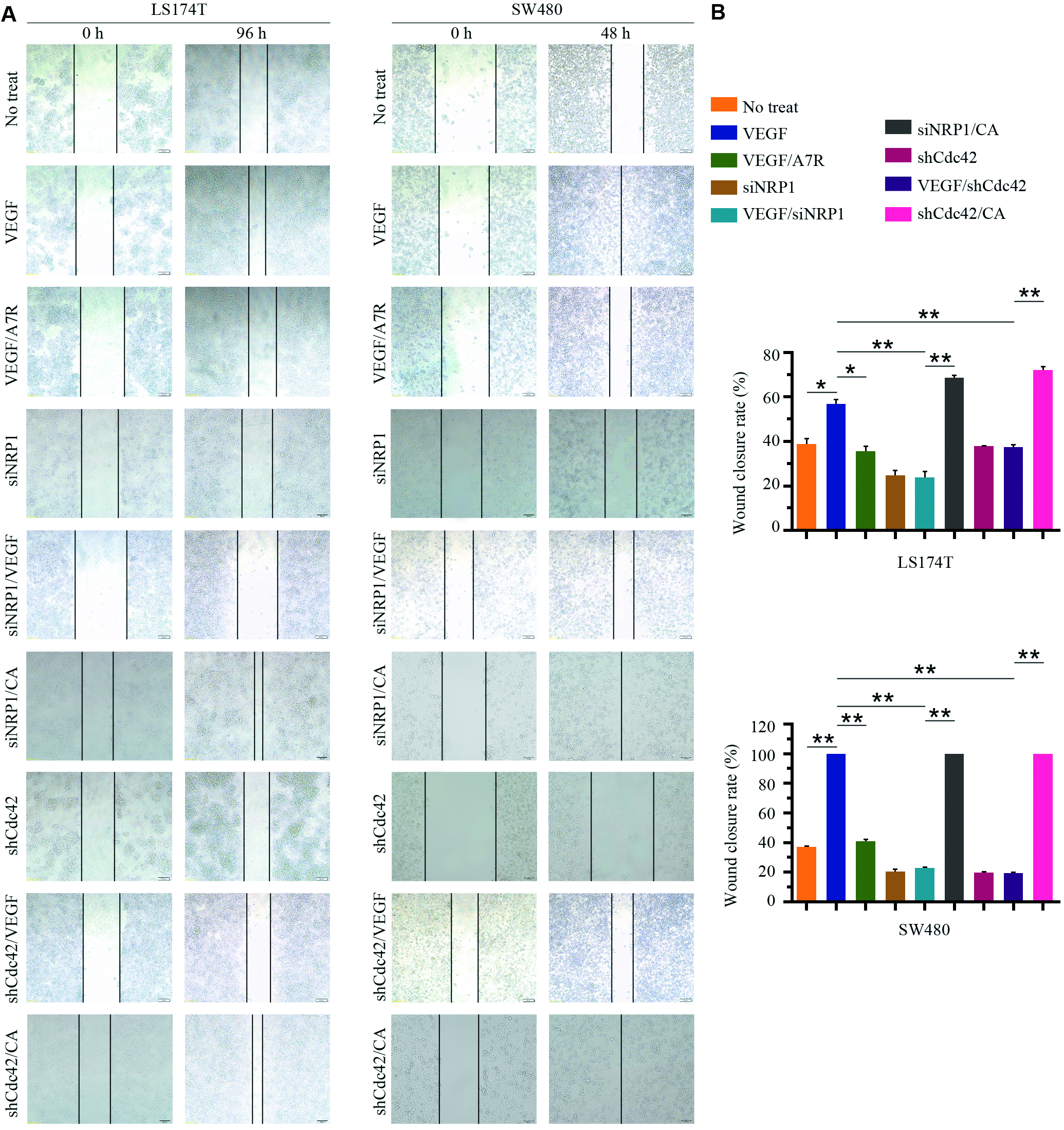

Supplement: Supplementary file 2 — Supplemental Fig. 1 [file 41419_2020_2370_MOESM2_ESM.tif]
