# Supplementary material for: Cdc42 subcellular relocation in response to VEGF/NRP1 engagement is associated with the poor prognosis of colorectal cancer
Source: Cell Death Dis. 2020 Mar 5;11(3):171. doi: 10.1038/s41419-020-2370-y (PMC7058620; doi:10.1038/s41419-020-2370-y)
Supplement: Supplementary file 3 — Author Contribution Form [file 41419_2020_2370_MOESM3_ESM.pdf]

DECLARATION OF CONTRIBUTIONS TO ARTICLE

ADMC

Manuscript Number: CDDIS-19-4108R

Journal Name: *Cell Death & Disease* (the 'Journal')

Proposed Title of the Contribution:

Cdc42 subcellular relocation in response to VEGF/NRP1 engagement is associated with the poor prognosis of colorectal cancer (the 'Contribution)

Author(s):

Li-li Ma, Li-li Guo, Yang Luo, Guang-long Liu, Yan Lei, Fang-yan Jing, Yun-li Zhang, Gui-hui Tong, Zhi-Liang Jing, Lan Shen, Min-shan Tang , Yan-qing Ding, Yong-jian Deng (the 'Authors')

For all *CDDis* articles, each person named as an author in the published version must be able to show he or she has contributed substantially to the article.

Authorship credit should be based on 1) substantial contributions to conception and design, acquisition of data, or analysis and interpretation of data; 2) drafting the article or revising it critically for important intellectual content; and 3) final approval of the version to be published. Authors should meet conditions 1, 2 and 3.

Any person who cannot be shown to have made a substantial contribution to the article cannot be listed as an author in the final version. The name of any person who is deemed to have made a minor contribution can, however, appear in the Acknowledgments section of the article.

Please complete the table below to indicate the contributions of all named authors to the manuscript.

Author Full Name:	Specification of Contribution to the Manuscript:
Yan-qing Ding, Yong-jian Deng, and Li-li Ma	Designed the study
Li-li Ma, Li-li Guo, and Yang Luo	Performed the cell experiments
Guang-long Liu, Yan Lei, and Yang Luo	Performed the western blot analysis
Lan Shen, and Gui-hui Tong	Performed immunohistochemical and immunofluorescence staining
Yun li Zhang, Zhi-liang Jing, Fang-yan Jing	Carried out clinical data collection and analysis
Min-shan Tang and Yang Luo	Helped with the animal studies
Li-li Ma and Yang Luo	Prepared the final manuscript

Please complete the table below to indicate the contributions of all named authors to the figures.

Figure 1:

Yan-qing Ding, Yong-jian Deng, and Li-li Ma designed the study;
Lan Shen, and Gui-hui Tong performed immunohistochemical staining;
Yun li Zhang, Zhi-liang Jing, Fang-yan Jing, carried out clinical data collection and analysis.

Figure 2:

Li-li Ma, Li-li Guo, and Yang Luo performed the cell experiments;
Guang-long Liu, Yan Lei performed the western blot analysis;
Lan Shen, and Gui-hui Tong performed immunofluorescence staining.

Figure 3:

Li-li Ma, Li-li Guo, and Yang Luo performed the cell experiments;
Guang-long Liu, Yan Lei, performed the western blot analysis;
Lan Shen, and Gui-hui Tong performed immunofluorescence staining.

Figure 4:

Li-li Ma, Li-li Guo, and Yang Luo performed the cell experiments;
Guang-long Liu, Yan Lei, performed the western blot analysis;
Lan Shen, and Gui-hui Tong performed immunofluorescence staining.

Figure 5:

Li-li Ma, Li-li Guo, and Yang Luo performed the cell experiments;
Guang-long Liu, Yan Lei, performed the western blot analysis;
Lan Shen, and Gui-hui Tong performed immunofluorescence staining.

Figure 6:

Li-li Ma, Li-li Guo, and Yang Luo performed the cell experiments;
Guang-long Liu, Yan Lei, performed the western blot analysis;
Min-shan Tang and Yang Luo helped with the animal studies;
Guang-long Liu, Yan Lei did the Transwell assays.

Signed for and on behalf of the Author(s):	Print Name:	Date:
Yongjian Deng	Yongjian Deng	2020/02/07
